# What factors indicate prognosis for adults with depression in primary care? A protocol for meta-analyses of individual patient data using the Dep-GP database

**DOI:** 10.12688/wellcomeopenres.15225.3

**Published:** 2020-04-01

**Authors:** Joshua E.J. Buckman, Rob Saunders, Zachary D. Cohen, Katherine Clarke, Gareth Ambler, Robert J. DeRubeis, Simon Gilbody, Steven D. Hollon, Tony Kendrick, Edward Watkins, Ian R. White, Glyn Lewis, Stephen Pilling

**Affiliations:** 1 Centre for Outcomes Research and Effectiveness (CORE), Research Department of Clinical, Educational & Health Psychology, University College London, London, WC1E 7HB, UK; 2 Department of Psychiatry, University of California, Los Angeles, Los Angeles, CA, 90095, USA; 3 Statistical Science, University College London, London, WC1E 7HB, UK; 4 School of Arts and Sciences, Department of Psychology, University of Pennsylvania, Philadelphia, PA, 19104-60185, USA; 5 Department of Health Sciences, University of York, York, YO10 5DD, UK; 6 Department of Psychology, Vanderbilt University, Nashville, TN, 407817, USA; 7 Primary Care & Population Sciences, Faculty of Medicine, University of Southampton, Southampton, SO16 5ST, UK; 8 Department of Psychology, University of Exeter, Exeter, EX4 4QG, UK; 9 Institute of Clinical Trials and Methodology, MRC Clinical Trials Unit, University College London, London, WC1V 6LJ, UK; 10 Division of Psychiatry, University College London, London, W1T 7NF, UK

**Keywords:** Depression, Prognosis, Primary Health Care, Meta-analysis, Individual Patient Data, Systematic Review, Protocol

## Abstract

**Background**: Pre-treatment severity is a key indicator of prognosis for those with depression. Knowledge is limited on how best to encompass severity of disorders. A number of non-severity related factors such as social support and life events are also indicators of prognosis. It is not clear whether this holds true after adjusting for pre-treatment severity as a) a depressive symptom scale score, and b) a broader construct encompassing symptom severity and related indicators: “disorder severity”. In order to investigate this, data from the individual participants of clinical trials which have measured a breadth of “disorder severity” related factors are needed.

**Aims**: 1) To assess the association between outcomes for adults seeking treatment for depression and the severity of depression pre-treatment, considered both as i) depressive symptom severity only and ii) “disorder severity” which includes depressive symptom severity and comorbid anxiety, chronicity, history of depression, history of previous treatment, functional impairment and health-related quality of life.

2) To determine whether i) social support, ii) life events, iii) alcohol misuse, and iv) demographic factors (sex, age, ethnicity, marital status, employment status, level of educational attainment, and financial wellbeing) are prognostic indicators of outcomes, independent of baseline “disorder severity” and the type of treatment received.

**Methods**: Databases were searched for randomised clinical trials (RCTs) that recruited adults seeking treatment for depression from their general practitioners and used the same diagnostic and screening instrument to measure severity at baseline – the Revised Clinical Interview Schedule; outcome measures could differ between studies. Chief investigators of all studies meeting inclusion criteria were contacted and individual patient data (IPD) were requested.

**Conclusions**: In total 15 RCTs met inclusion criteria. The Dep-GP database will include the 6271 participants from the 13 studies that provided IPD. This protocol outlines how these data will be analysed.

**Registration**: PROSPERO CRD42019129512 (01/04/2019)

## Introduction

One in 20 adults across the globe will experience an episode of major depression every year ( Thornicroft *et al*., 2017), most of whom will not receive any treatment ( Olfson *et al*., 2016; Thornicroft *et al*., 2017). For those that do get treatment the majority will either not reach remission or it will take a number of trials of different treatments before they do ( Kessler, 2018; Rush *et al*., 2006). Not reaching full remission is one of the strongest predictors of relapse and recurrence ( Buckman *et al*., 2018). There is a lack of knowledge of prognosis independent of treatment and within different types of treatment, and therefore a lack of evidence with which to make informed choices of whether any active treatment should be trialled, or which type of treatment to trial at any given point, for any given individual (e.g. Cohen & DeRubeis, 2018).

In order to reduce the burden of depression it is imperative that we understand more about the response to treatments, and their limits, to better consider the risk for poor prognostic outcomes. One major focus has been on the effect of baseline severity on outcomes. That severity is related to outcome holds with the ‘common-sense’ view of most illnesses, depression included, but as recommended by Leucht and colleagues ( Leucht *et al*., 2015) the consideration of prognosis needs to account for more than just the number or intensity of depressive symptoms. Studies considering the role of pre-treatment depressive severity have typically been limited to group level analyses (e.g. Kirsch *et al*., 2008) so have been unable to consider severity beyond a score on a depressive symptom measure. In so doing, these studies have been unable to account for the seriousness of the presentation of depression (e.g. Leucht *et al*., 2012)). Such studies and others that have utilised individual patient data have also typically been limited to a narrow band of treatment types (e.g. Fournier *et al*., 2010) and to studies with small sample sizes (e.g. Cohen *et al*., 2019), limiting their generalizability ( Rothwell, 2005).

Several factors which may be considered in conjunction with depressive symptom scale scores as part of “disorder severity”, and could potentially act through the same mechanisms on outcome (e.g. Fried & Nesse, 2014), have also been found to be important in prognostic models. For example: factors related to past experiences of depression, duration or chronicity ( Fournier *et al*., 2009), a history of depression (e.g. Chekroud *et al*., 2016), and a history of previous treatments for depression (e.g. DeRubeis *et al*., 2014); and functional impairment (e.g. Delgadillo *et al*., 2016; Saunders *et al*., 2016) are all indicators of prognosis.

There is a lack of agreement on the prognostic role of anxiety symptoms and of comorbid anxiety disorders for those with depression despite agreement that symptoms of anxiety are common among those with depression either as part of their depressive episode or another comorbid disorder (e.g. Kessler *et al*., 2005; Sartorius *et al*., 1996). Somatic anxiety and avoidance related symptoms of agoraphobia ( Chekroud *et al*., 2016) have been found to be prognostic for those treated with antidepressant medications (ADM) but not in those treated with psychological therapies (e.g. Lutz *et al*., 2006). Symptoms of generalised anxiety disorder and phobias have also been found to be predictive of outcomes in some clinical cohorts ( Saunders *et al*., 2019; Saunders *et al*., 2016); but not in others ( Delgadillo *et al*., 2016). Given the high rates of comorbidity and the co-occurrence of depressive and anxious symptoms even at sub-clinical levels, it would be useful to know whether the prognostic effects of anxiety symptoms and disorders (collectively or individually) operate independently from depressive symptom severity, “disorder severity”, and independent of the type of treatment given, if any. One potential explanation for the somewhat contradictory findings on the role of anxiety symptoms and disorders on the prognosis of patients with depression is that many studies have used different scales to measure the same and indeed different anxiety conditions. Consistency in the measurement of such factors might allow for a more definitive investigation of the prognostic role of such symptoms and disorders.

There is similar disagreement regarding alcohol misuse as an indicator of prognosis, it is also highly comorbid with depression but has been less well studied (e.g. Weaver *et al*., 2003). Some studies have suggested that alcohol misuse (excluding alcohol dependence) is a prognostic indicator of treatment outcomes for those with depression ( Clarkson *et al*., 2016), but others have suggested that it is unrelated to treatment outcomes ( Boschloo *et al*., 2012) and instead is predictive only of dropping out of treatment ( Buckman *et al*., 2018). There are several other factors that may be related to depression treatment outcomes but again, the effects have been less well studied. These include health-rated quality of life (e.g. Huibers *et al*., 2014), social support (e.g. Hallgren *et al*., 2017) and life events recent to the present episode (e.g. DeRubeis *et al*., 2014; Fournier *et al*., 2009). This leaves the question then of whether or not these factors are indicative of prognosis independent of baseline severity (whether this encompasses only depressive symptoms (“symptom severity”) or the wider construct of “disorder severity” including other factors noted above), and independently of treatment. In addition, a number of demographic factors have been found to be important in predictive models of depression outcomes alongside symptom severity, including: age (e.g. Delgadillo *et al*., 2016; Fournier *et al*., 2009); gender ( Saunders *et al*., 2016); ethnicity (e.g. Chekroud *et al*., 2016; Saunders *et al*., 2016); marital status (e.g. Fournier *et al*., 2009); employment status (e.g. Chekroud *et al*., 2016; Fournier *et al*., 2009); level of educational attainment (e.g. Chekroud *et al*., 2016); and markers of socio-economic status or financial stability/security (e.g. Saunders *et al*., 2016). However, whether these factors are indicators of prognosis independent of severity (either as just depressive symptoms or the wider construct encompassing more than just depressive symptoms) remains to be seen. Here, we use the phrase independent of treatment to highlight that we wish to investigate factors that affect outcome regardless of any treatments rather than trying to identify factors that help predict response to a given type of treatment or those that predict differential response to two or more treatments.

Over the last three years a number of authors of the current article have worked to collect individual participant data (IPD) from randomised clinical trials (RCTs) of any treatment for depression, recruited from primary care services/general practice, that used the same clinical interview schedule (the CIS-R) to measure “disorder severity” factors, determine diagnoses, and capture symptoms across a range of depressive and anxious disorders. This article explains how that IPD dataset was formed and describes a protocol for a series of analyses of it.

## Aims and objectives

1) To determine whether certain “disorder severity” factors are indicators of prognosis, independent of treatment, and independent of baseline depression symptom scale scores. These are i) chronicity of depression at baseline; ii) a history of depression; iii) a history of any previous treatment for depression; iv) a history of ADM treatment; v) anxiety symptom severity; vi) presence of and number of comorbid anxiety disorders; vii) duration of anxiety problems; viii) functional impairment; and ix) health-related quality of life.
2) To determine whether or not the following are indicators of prognosis independent of severity of depression as measured in both ways outlined in 1 above - symptom severity, and “disorder severity”:
  i) social support
  ii) the occurrence of recent stressful life events
  iii) alcohol misuse
  iv) demographic factors (age, gender, ethnicity, employment status, marital status, highest level of educational attainment, and financial wellbeing including housing status)

## Methods

### Identification and selection of studies

Studies were identified using a combination of keyword and subject heading searches on the bibliographic databases below, hand-searching through the references of studies identified in the searches, and by contacting experts for unpublished or missed studies. Searches were run in several stages, firstly to scope the literature in November 2015 and in order to refine inclusion and exclusion criteria, again in April 2016 to identify studies and begin the process of data collection, then finally in March 2019 to ensure no studies published more recently were missed. The final searches were run on the Cochrane CENTRAL Trial Register (searched on 20 ^th^ March 2019), Embase 1947 to 2019 Week 12, International Pharmaceutical Abstracts 1970 to March 2019, Ovid MEDLINE 1946 to March Week 3 2019, and PsycINFO 1806 to March Week 3 2019. Search terms included variations of phrases such as “depression” or “major depression”, “RCT” or “Randomised Controlled Trial” or “Clinical Trial”, and “CIS-R” or “Clinical Interview Schedule”. Full details of the searches are provided as Extended data ( Buckman, 2019).

A single reviewer (JB) screened titles and abstracts of potentially eligible studies returned by the searches, those that were potentially relevant to the review were then read in full and judged against inclusion/exclusion criteria. Uncertainties in inclusion/exclusion were discussed with two other reviewers (GL and SP). Relevant studies were then read in full by all three reviewers before reaching consensus.

### Inclusion & exclusion criteria

Inclusion and exclusion criteria were refined over the stages of running scoping searches for this work. After this refining process, studies were included if they were randomised clinical trials (RCTs) of adults (aged 16 or over), had at least one active treatment arm, and used the CIS-R at baseline to measure symptoms of anxiety and depression and to determine diagnoses. The study samples had to have unipolar depression, depressive symptoms significant enough to lead them to seek treatment from their GP, or a CIS-R ( Lewis *et al*., 1992) score of ≥12; recruited from primary care centres. While all studies had to use the CIS-R at baseline, outcome measures could differ between studies.

Studies were excluded if they did not meet the above criteria and if they: included patients with depression as a secondary diagnosis in studies of adults with personality disorders, psychotic conditions, or neurological conditions; were studies of adults with bi-polar or psychotic depressions; were studies of children or adolescents; were feasibility studies only; or did not recruit participants from General Practices or in primary care.

### Measures

The relevant measures included in the identified studies are:

The CIS-R ( Lewis *et al*., 1992): consists of 14 symptom subsections scored 0–4 covering core features of depression, depressive thoughts (scored 0–5), fatigue, concentration/forgetfulness, and sleep, generalized anxiety, worry, irritability, obsessions, compulsions, health anxiety/somatic concerns, phobic anxiety (split into agoraphobia, social phobia, and specific phobia), and panic. A final section measures general health, impairment and weight change. The total score ranges from 0–57 with a cut-off of ≥12 used to indicate likely common mental disorder, primary and secondary diagnoses using ICD-10 criteria are given as are binary indictors of diagnosis for all the disorders assessed.

Beck Depression Inventory (BDI-II: Beck *et al*., 1996)): used to measure depressive symptoms, each item is scored 0–3 with a maximum score obtainable of 63. A cut-off of ≥10 is used indicate significant symptoms of depression.

Patient Health Questionnaire (PHQ-9: Kroenke *et al*., 2001) is a 9-item depression screening measure. Items are scored 0–3, a cut-off of ≥10 is used to indicate “caseness” for depression.

Hospital Anxiety and Depression Scale (HADS: Zigmond & Snaith, 1983): measures symptoms on two subscales, depression and anxiety. The cut-off for caseness on the depression subscale is ≥8.

General Health Questionnaire (12-item version) (GHQ-12: Goldberg, 1992): a cut-off of ≥2 is used to indicate the presence of common mental disorders.

Edinburgh Postnatal Depression Scale (EPDS: Cox *et al*., 1987)): measures symptoms of depression focussed on women in the post-natal period, scores of ≥13 are indicative of a depressive episode

Social Support: an 8-item instrument assessing the degree to which participants rated the social support of their friends and family in each of the following domains: 1) being accepted for who one is; 2) feeling cared about; 3) feeling loved; 4) feeling important to them; 5) being able to rely on them; 6) feeling well supported and encouraged by them; 7) being made to feel happy by them; and 8) feeling able to talk to them whenever one might like. These were adapted by authors of RCTs (e.g. Kessler *et al*., 2009) included in this IPD from items of the Medical Outcomes Study Social Support Survey: ( Sherboune & Stewart, 1991). Items are scored 1–3, with total scores ranging from 8–24; higher scores indicate higher levels of perceived social support.

Life events: the Social Readjustment Rating Scale ( Holmes & Rahe, 1967): participants are asked to say yes/no to whether they have suffered any of nine events within the last six months e.g. a death/bereavement; being physically attacked/injured; or going through a divorce/separation. Each item is scored yes (1) or no (0) and the total score is the sum of all the items.

Alcohol use: the alcohol use disorder identification test primary care version (AUDIT-PC: Piccinelli *et al*., 1997) was used to assess alcohol misuse, this includes five items scored 0–4. A cut-off of ≥5 indicates hazardous alcohol use that may be harmful to one’s health.

Health related quality of life: EQ-5D-3L & EQ-5D-5L: ( Herdman *et al*., 2011)): the EQ-5D is a generic measure of health status in five domains – mobility; self-care; usual activities; pain/discomfort; and anxiety/depression. Each domain in the 3L version has three response categories ranging from no problem present (1) to extreme problems in the given domain (3), the 5L version has five response options ranging from “I have no problems…” (1) to “I am unable to…” or “I have/am extreme/extremely…” (5). A total score is derived from summing the score on the five items with higher scores indicating more severe health problems than lower scores. A cross-walk of scores from the 3L and 5L versions will be used to derive a continuous index score representing the EQ-5D total score in the present study ( van Hout *et al*., 2012).

### Characteristics of the included studies

**Figure 1.**
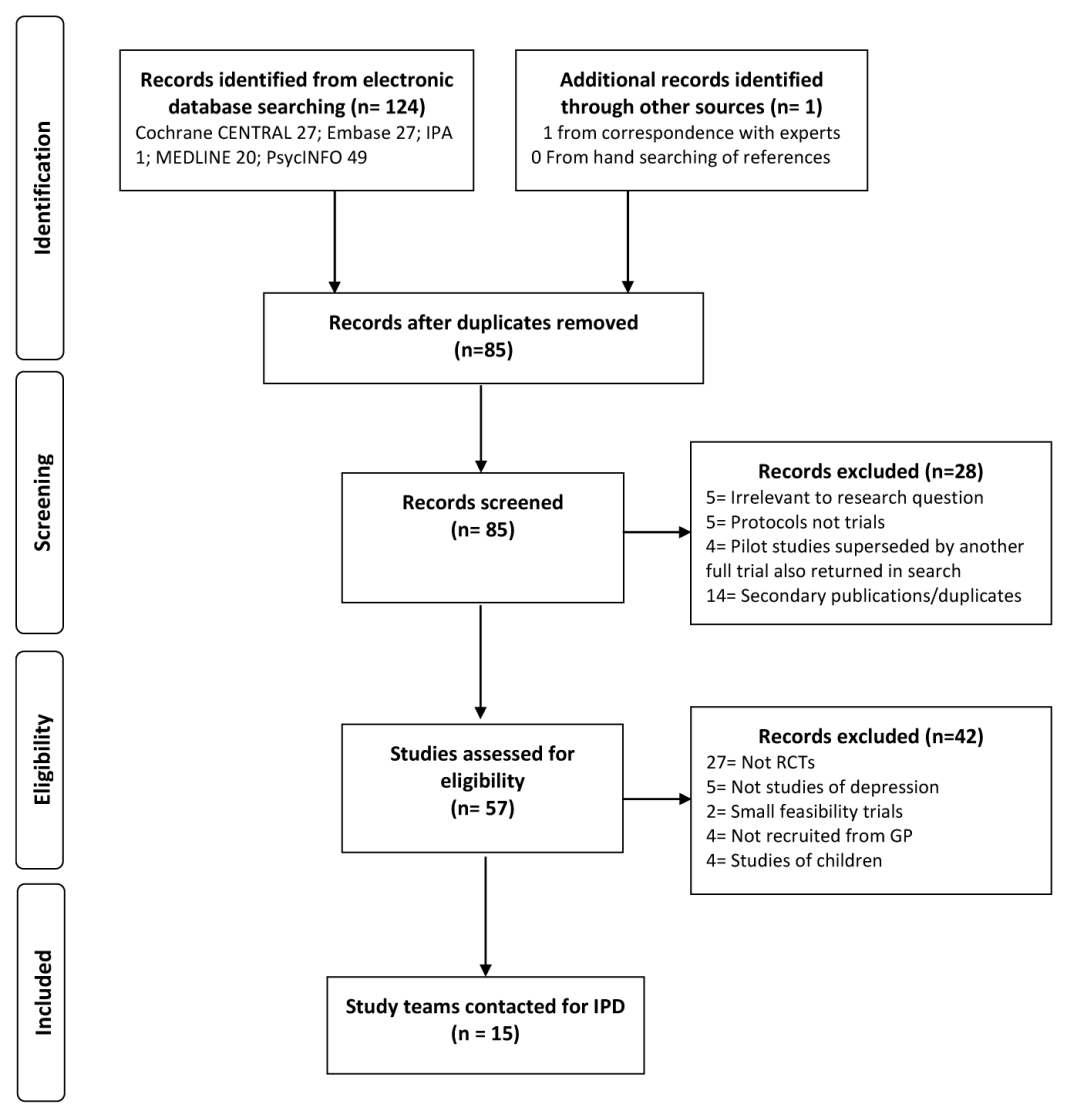
Flow diagram of study selection.

In total, 15 RCTs were identified as meeting inclusion criteria for the IPD, of which 12 have provided individual patient-data and one is in the process of providing these data, see Figure 1. Nine studies were identified at the initial scoping stage, with a further three found in the second stage of searching and one final further study identified as meeting inclusion criteria after the final searches. After a final consensus meeting once the final searches had been run, two studies previously considered to not meet inclusion criteria were re-classified as meeting criteria, and the study authors were contacted for IPD accordingly. A description of each included study for which authors agreed to provide IPD can be found in Table 1 and descriptive statistics and degrees of missingness for key predictor and outcome variables discussed below are presented in the Extended data ( Buckman, 2019). Integrity of the data for each study was checked with the study team and against details published about each study, discrepancies were discussed and investigated in conjunction with each study team until satisfactory explanations were found and updated data were provided if appropriate and if required.

**Table 1.** Description of studies included in individual patient data (IPD).

Study	N at T0	Sample	Interventions	Outcome Measure	Endpoint at 3–4 months	N at 3–4 months
AHEAD ( Kendrick *et al*., 2006)	327	Adults with new depressive episodes diagnosed by GP	TCA vs SSRI vs Lofepramine	HADS (also CIS-R)	Yes	169
CADET ( Richards *et al*., 2013)	527	Adults ≥18, ICD-10 Depressive Episode	Collaborative Care vs TAU	PHQ-9	Yes	505
COBALT ( Thomas *et al*., 2012)	469	Adults 18–75 with treatment resistant depression, scoring ≥14 BDI-II	TAU vs CBT+TAU	BDI-II (also PHQ-9)	Yes	469
CPN-GP ( Kendrick *et al*., 2005; Kendrick *et al*., 2006)	247	Adults 18–65 seeking treatment from GP for new episode of depression, anxiety or reaction to life difficulties, scoring >3 on GHQ-12 with between 4weeks and 6months duration	TAU vs CPN vs CPN + Problem solving	CIS-R (also HADS)	No	210
GENPOD ( Lewis *et al*., 2011)	601	Adults 18–74 with depressive episode	Citalopram vs Reboxetine	BDI-II (also HADS)	Yes	601
HEALTHLINES ( Salisbury *et al*., 2016)	609	Adults ≥18, PHQ-9 score ≥10, confirmed diagnosis of depression with CIS-R, internet access	TAU vs Healthlines telecare + TAU	PHQ-9	Yes	525
IPCRESS ( Kessler *et al*., 2009)	295	Adults scoring ≥14 BDI-II and GP confirmed diagnosis of depression	iCBT vs TAU	BDI-II	Yes	299
ITAS ( Thomas *et al*., 2004)	798	Adults ≥16, scored ≥12 on CIS-R	TAU vs Recommendation + TAU	GHQ-12	No	617
MIR ( Kessler *et al*., 2018)	480	Adults ≥18 taking SSRIs or SNRIs at adequate dose for≥ 6 weeks, and scored ≥14 on BDI-II	Mirtazapine vs Placebo	BDI-II (also PHQ-9)	Yes	424
PANDA ( Salaminios *et al*., 2017)	652	Adults presenting with low mood or depression to GP in last 2 years, free of ADM for 8 weeks up to baseline	Sertraline vs Placebo	PHQ-9 (also BDI-II)	Yes	653
REEACT ( Gilbody *et al*., 2015)	685	Adults with PHQ-9>10 presenting to GP with depression	Moodgym vs Beating the Blues vs TAU	PHQ-9	Yes	526
RESPOND ( Sharp *et al*., 2010)	220	Women meeting criteria for MDD within 6-months post- partum	ADM vs Listening intervention	EPDS	Yes	218
TREAD ( Chalder *et al*., 2012)	361	Adults 18–69 who met diagnostic criteria for MDD and scored ≥14 on BDI-II	TAU vs Physical Activity + TAU	BDI-II	Yes	290

Abbreviations: ADM – antidepressant medication; BDI-II – Beck Depression Inventory; EPDS – Edinburgh Postnatal Depression Scale; GHQ-12 – General Health Questionnaire 12 item version; HADS-D – Hospital Anxiety and Depression Scale – depression subscale; iCBT (internet based therapist delivered cognitive behavioural therapy); MDD – Major Depressive Disorder; T0 - Baseline; TAU – treatment as usual; TCA – tricyclic antidepressant

## Ethical considerations and trial registrations

All studies included in the Dep-GP database were granted ethical approvals by NHS Research Ethics Committees. Specific ethical approvals and trial registration details are given in Extended data ( Buckman, 2019).

### Data analysis plan

***End-point data.*** Of the included studies, 11 collected endpoint data between three and four months post-baseline (see Table 1), this will be the primary endpoint of interest for the analyses outlined in this protocol Additional end-points between six and eight months, and nine and 12 months post-baseline will be used for sensitivity analyses (see Table 2). Endpoints prior to three months or after 12 months will be excluded from the present analyses.

**Table 2.** Endpoints and time from baseline in weeks in each study in the Dep-GP database.

	Endpoint and time from baseline in weeks (w)		
	3–4m	6–8m	9–12m
Study	12–18w	24–32w	36–52w
AHEAD	12w	26w	52w
CADET	16w		52w
COBALT	12w	26w	36w
CPN-GP		26w	
GENPOD	12w		
HEALTHLINES	16w	32w	52w
IPCRESS	16w	32w	
ITAS		26w	
MIR	12w	24w	52w
PANDA	12w		
REEACT	16w		52w
RESPOND	18w		44w
TREAD	16w	32w	52w

### Outcomes

***Primary outcomes.*** The primary outcome for the present analyses will be the score on the primary depressive symptom measure used at 3–4 months post-baseline. Scores on the different measures of depressive symptoms used across the studies at the endpoints will be z-score standardised. This will be done for each symptom measure using the mean and standard deviation (SD) at 3–4 months pooled across all arms of all studies that reported that symptom measure at that time. The same mean and SD will be used to create z-scores for secondary outcomes at 6–8 and 9–12 months post-baseline.

A second primary outcome will be the log of 3–4 month post-baseline depression scale scores, without standardising across the measures. This will allow for the consideration of proportional change in symptom scores (e.g. Button *et al*., 2015)

***Secondary outcomes.*** In any analysis where the only outcome measures used in the studies of the Dep-GP database were the BDI-II or the PHQ-9 a secondary outcome will be a conversion of those two measure scores to the PROMIS T-score ( Choi *et al*., 2014). This will be achieved using cross-walk tables derived from an item-response theory based analysis of several depression symptom measures ( Choi *et al*., 2014).

Additional secondary outcomes will be partial remission on each of the primary outcome measures used in each study (scores below the cut-off for caseness on each measure as described in Measures section above), and the proportion of participants that dropped-out/withdrew from each study at each time-point.

### Prognostic indicators under consideration

1. “Disorder severity” of depression at baseline, from self-reported:
  • scores on the depressive symptom measures detailed above
  • the sum of the scores on the depressive sub-scales of the CIS-R
  • the sum of the scores on the non-depressive/anxiety sub-scales of the CIS-R combined, and individually by subscale
  • the number and type of comorbid anxiety disorders
  • the duration of depression
  • the duration of anxiety
  • whether or not participants have a history of depression
  • whether or not participants have a history of previous treatment for depression, and whether or not participants have a history of ADM treatment
  • whether or not participants were experiencing significant functional impairment at baseline
  • Health-rated quality of life at baseline.

2. Demographic factors
  • Age
  • Gender
  • Ethnicity
  • employment status
  • marital status
  • highest level of educational attainment
  • financial wellbeing
  • housing tenure

3. Social support in all eight domains listed in the Measures section above and the total score on the measure.
4. Life events in all nine domains as discussed in the Measures above and the total score.
5. Total score on the alcohol measure

### Confounding factors

Different confounding factors will be considered in relation to each prognostic factor under investigation. Determinations of which factors to include in the meta-analytic models as confounders will be made based on *a priori* considerations of the relationship under investigation and the relationships between the confounder and both the prognostic indicator and outcome. Only factors that are independently associated with both the prognostic factor and the outcome, are not potentially caused by the prognostic factor, and affect the association between the prognostic factor and outcome will be considered as potential confounders. For example, age is *a priori* assumed to confound the relationship between duration of depression and outcome at 3-to-4 months. The presence of any long-term physical health condition might be considered a confounder in the relationship between health-related quality of life and outcome. In addition, research site or centre, and the clinical and demographic factors listed above in the prognostic indicator section (for analyses in which they are not the predictor of interest) will all be investigated as potential confounders. The variables used to stratify the randomisation beyond site and initial depressive symptom severity will be investigated as potential confounders within each study. Treatment allocation, i.e. the randomisation in each study will be controlled for in all multivariable models.

## Data handling and data management

### Pre-processing

Data from the 12 trials were received and cleaned on an individual study basis before combining all studies into a single aggregated dataset, the final Dep-GP dataset will be formed once data from the 13 ^th^ study are received and cleaned.

A number of baseline variables were re-categorised into higher-order categories due to small numbers, see Table 3. Of note, there was poorer data-coverage across the IPD on information about the number of past depressive episodes than there was on a separate question about whether or not the participant had any previous episodes, see Extended data ( Buckman, 2019).

**Table 3.** Categorisation of variables during data pre-processing.

Variable	Original categories	New categories
**Ethnicity**	White	White
	Mixed	Other
	Black	
	Asian	
	Chinese	
	Other	
**Employment Status**	Full time employed	Employed
	Part time employed	
	Student	Not seeking employment
	Retired	
	House-person	
	Other	
	Unemployed jobseeker	Unemployed
	Unemployed due to ill-health	
**Marital Status**	Married/cohabiting	Married/cohabiting
	Single	Single
	Separated	No longer married
	Divorced	
	Widowed	
**Highest level of education**	Degree or higher	Degree or higher
	Foundation Degree/Diploma	A-level or Diplomas
	A-level	
	GCSE	GCSE
	Other qualifications	None or Other
	No formal qualifications	
**Financial Wellbeing**	Living Comfortably	OK financially
	Doing alright	
	Just about getting by	Just about getting by
	Hard to make ends meet	Struggling financially
	Very hard to make ends meet	
**Long-term Health** **Condition Status**	None	No long-term physical health conditions
	Mental Health Only	
	Diabetes	At least one long-term physical health condition
	Asthma or COPD	
	Arthritis	
	Heart Disease	
	Stroke	
	Cancer	
	Kidney Disease	

Further pre-processing for the analyses specified below will be considered. The distributions of all variables will be inspected prior to imputation (discussed further below). Continuous variables that are non-normally distributed will be transformed to normality prior to imputation. If transformation is required of the prognostic indicators these will only be log transformed in order that the interpretation of their effects is sensible. If log-transformation does not result in approximate normality of the distribution of these variables, predictive mean matching ( Morris *et al*., 2014) will be used for imputation of missing data as part of the multiple imputation with chained equations approach discussed further below.

### Missing data

Missing data will be imputed using multiple imputation with chained equations (MICE) in Stata 15.0 ( StataCorp, 2017). This approach uses regression models to impute missing values. A number of imputed datasets (here we will use 50) are produced to reflect the uncertainty/variability in the imputation process. If data are not reasonably able to be log transformed to meet normality assumptions, predictive mean matching (PMM) via a k-nearest neighbours approach will be used as it is considered to be more appropriate for non-normal continuous variables ( Horton & Lipsitz, 2001), here we will use k=10. Linear regression will be used for approximately normally distributed continuous variables, logistic regression models will be used for binary variables, and ordinal and multinomial regression models will be used for ordered and unordered categorical variables respectively. All imputation models will be built using data on baseline and outcome variables following conventions (e.g. Royston & White, 2011). Only variables with less than 50% missing data will imputed (see Extended data for degrees of missing by variable ( Buckman, 2019)). All imputation models will be run to produce 50 imputed datasets. If the primary analysis (detailed below) shows that results differ considerably when studies with systematically missing baseline data are included/excluded from the meta-analytic models, then a separate imputation approach will be taken to impute these systematically missing data: multiple imputation with multilevel random effects for study (e.g. Resche-Rigon *et al*., 2013).

### Software & packages

Stata SE 15 ( StataCorp, 2017): ipdmetan ( Fisher, 2015) and ICE ( Royston, 2009), mi impute pmm ( Morris *et al*., 2014) packages.

### Primary analyses

To investigate Aim 1 linear regression models of the score on the depressive symptom scales at 3–4 months post-baseline will be built in each study, adjusting for the random allocation in each study, baseline depression scale scores in each study, and then separately for other “disorder severity” related factors listed above. Estimates from each study will then be pooled in random effects meta-analyses. A multivariable model of outcome will be built considering all of the “disorder severity” factors that are significantly associated with outcome after adjusting for baseline depressive symptom scale scores alone. This will be done initially with only variables that are not systematically missing between the studies, such models will be built firstly on all studies and then on all studies that do not have systematically missing covariates that could otherwise have been included in the multivariable model. These models will be compared and if there is a considerable difference in the effects systematically variables will then be imputed as described above. Decisions on which factors to include/exclude in the multivariable models will be led by consideration of the unique contribution to the models by each variable, the amount of variance explained (R ^2^) when modelled with and without the given factor, and to tests of the assumptions of linear regression models. If there are high degrees of multicollinearity the variable(s) explaining most variance in outcome will be retained in the model while the other(s) is/are removed. Link tests will be performed to consider the appropriateness of the linear link function. Multivariate normality, homoscedasticity, and overly influential data points will be considered by plotting residuals, and assessing Cook’s distance in the residuals plotted against leverage.

Aim 2 - Separate meta-analyses will be conducted with each of the prognostic indicators under consideration (social support, life events, alcohol misuse, and the demographic factors outlined in the Introduction above), unadjusted and adjusted for severity (symptom severity and “disorder severity”) to determine whether or not they are indicative of outcome of treatment independently from either or both of symptom severity and “disorder severity”.

There will therefore be three models of the primary outcome built for each prognostic factor assessed and an additional model just for the confounding factors and the baseline depressive symptom scale scores:

1. Baseline depressive symptom scale score adjusted for confounding factors.
2. As in 1 but with the addition of each “disorder severity” factor (one by one).
3. As in 1 with the addition of all “disorder severity” factors that were significant or otherwise important in 2, and then removing factors that are no longer significant.
4. As in 3 with the addition of the other potential prognostic factors (e.g. social support) (one by one).

Meta-analyses will be conducted using the “ipdmetan” package in Stata ( Fisher, 2015) and displayed using forest plots. All meta-analyses will be conducted using a DerSimonian and Laird random effects model. This takes into account heterogeneity of coefficients between trials. The degree of heterogeneity will be assessed using prediction intervals and its impact will be assessed using the I ^2^ statistic ( Higgins *et al*., 2003).

### Secondary and sensitivity analyses

If heterogeneity between the studies were considerable based on guidance from the Cochrane Collaboration e.g. with I ^2^ above 75% or where the effect in one study appears to be considerably different from that of all other studies after inspecting the forest plot ( Higgins *et al.*, 2003), sensitivity analyses will be performed removing studies contributing most to the heterogeneity from the meta-analyses to consider their impact on the summary statistics. If the same variables were found to have considerable amounts of heterogeneity when analysed in each of the four models above, sensitivity analyses would be conducted for the model controlling for the most other variables, e.g. symptom severity and covariates (model 3) only. In addition, for variables in the final model(s), sensitivity analyses were similarly planned where the threshold for substantial heterogeneity was met (I ^2^ above 50%) ( Higgins *et al.*, 2003). Additional investigations of potential heterogeneity between the studies will involve assessing effects in Aim 1 in subgroups of patients including those with treatment resistant depression compared to those with a first episode and those with no history of treatment. Further sensitivity analyses will be conducted using the endpoint at 6-to-8 months in bivariate meta-analyses in order to include the two studies that did not have an endpoint in the 3-to-4 month post-baseline time period. This will initially be done only to assess the prognostic indication of baseline depressive scale scores adjusted for the confounding factors specified. If it is found that this leads to considerable variation in the results then this method will be similarly used in the analyses of the other potential prognostic factors.

In addition to considering the associations between social support and outcome as modelled with the total score on the social support scale, analyses will be conducted with each of the eight domains measured on that scale. Likewise, each of the nine domains measured in the life events scale will be considered individually.

### Sample size and power

The sample size for each of the proposed analyses will be dependent on the number of studies identified as relevant to that analysis and the degree of systematically missing data across the studies on the variables of interest. However, in Dep-GP there will be sufficient power to detect effects in all of outlined analyses as sample sizes will be beyond the minimum required to detect such effects. For example, for 80% power to detect an effect of depressive symptom severity of the same size found in a prior analyses (R ^2^ of 0.09: Delgadillo *et al*., 2017) with alpha set at 0.05, the minimum required sample size is 161 participants. It would be 105 participants to detect a similar effect for that found for employment status (R ^2 ^of 0.137). In Dep-GP there are data on 4679 participants at 3–4 months post-baseline or 5226 once missing outcome data have been imputed. These sample sizes would give 80% power to detect effects where R ^2^ is greater than or equal to 0.0033 or 0.0029 respectively, with alpha set a 0.05.

### Risk of bias

Risks of bias assessments will be conducted using the Quality in Prognosis Studies (QUIPS) ( Hayden *et al*., 2013). Two reviewers (JB & RS) will independently rate the risk of bias on the QUIPS in each study related to : i) study participation; ii) study attrition; iii) prognostic factor measurement; iv) outcome measurement; v) study confounding; and vi) statistical analysis and reporting. Studies well then be given a rating of “high risk”, “moderate risk” or “low risk”. The quality of evidence for each prognostic indicator will be assessed using the Grading Recommendations, Assessment, Development and Evaluations (GRADE) framework ( Guyatt *et al*., 2008).

## Discussion and conclusions

Knowledge of prognosis for those seeking treatment for depression after accounting for baseline severity has been limited to the consideration of severity only as a depressive symptom scale score, but many other related factors including the chronicity of depression and comorbid symptoms of anxiety have been found to be important prognostic indicators. In addition, a number of factors have been reported to be indicators of prognosis for depressed patients, but whether this is true after adjusting for severity encompassed in a scale score (symptom severity) or a more broad range of related factors (which here we call “disorder severity”) remains to be seen. In order to investigate this, data from the individual participants of a wide range of clinical trials which have measured this breadth of severity related factors is needed.

We found 15 studies that do this and met inclusion criteria, 12 have given IPD data and one is in the process of providing IPD data to help form the Dep-GP database, data from the remaining two studies were no longer available as these were conducted approximately 20 years ago. We will use differing subsets of the 13 studies to meet our aims as necessary where data on key variables are available. The consistency in setting and the variability in both the populations drawn upon in the 13 studies and the treatments received in those studies means that findings from the Dep-GP database may be generalizable to adults with depression seeking treatment from their GP/family physician.

### Study status

This is protocol version 1.3 last amended, 24 ^th^ October 2019. Data collection from eligible studies started in April 2016, agreement for data sharing from the final eligible study able to provide IPD was provided on 12 ^th^ March 2019 in principle and in full on 16 ^th^ September 2019, complete data for that study have not been provided yet. Estimated time to complete the outlined analyses is six months from the point at which we receive the final study dataset or if there is any reason that it cannot be provided, six months from the time we are notified of that eventuality.

### Dissemination

Findings from the analyses outlined above will be disseminated through peer-reviewed publications, and academic conference proceedings, through online blogs and other grey-literature and to appropriate service user research advisory groups linked to the host organisation.

## Data availability

### Underlying data

No data are associated with this article

### Extended data

Open Science Framework: What factors indicate prognosis for adults with depression in primary care? https://doi.org/10.17605/OSF.IO/UX95Q ( Buckman, 2019)

This project contains the following extended data:

- details of missing data across dep-gp studies.docx (Missing data from included studies)
- Ethics approval and trial registration details for dep-gp studies.docx (Ethics approval and trial registration details of included studies)
- Search results_OSF.docx (Search terms and results of searches)

### Reporting guidelines

Open Science Framework: PRISMA-P checklist ( Shamseer *et al*., 2015) for “What factors indicate prognosis for adults with depression in primary care? A protocol for meta-analyses of individual patient data using the Dep-GP database”. https://doi.org/10.17605/OSF.IO/UX95Q ( Buckman, 2019)

Data are available under the terms of the Creative Commons Zero “No rights reserved” data waiver (CC0 1.0 Public domain dedication).
