# Supplementary material for: What factors indicate prognosis for adults with depression in primary care? A protocol for meta-analyses of individual patient data using the Dep-GP database
Source: Wellcome Open Res. 2020 Apr 1;4:69. Originally published 2019 Apr 16. [Version 3] doi: 10.12688/wellcomeopenres.15225.3 (PMC6880263; doi:10.12688/wellcomeopenres.15225.3)
Supplement: Peer review history

# Peer review: Reviewer response for version 2

**DOI:** 10.21956/wellcomeopenres.17017.r36881

**Published:** 2019-11-21

**Authors:** Julieta Galante

**Affiliations:** 1 Department of Psychiatry, University of Cambridge, Cambridge, UK

I thank the authors for carefully considering my comments to their first manuscript. The revised version reads clearer to me and I have no further comments to make. I look forward to the results of this interesting project.

I confirm that I have read this submission and believe that I have an appropriate level of expertise to confirm that it is of an acceptable scientific standard.

# Peer review: Reviewer response for version 1

**DOI:** 10.21956/wellcomeopenres.16615.r35662

**Published:** 2019-10-23

**Authors:** Wolfgang Lutz, Brian Schwartz

**Affiliations:** 1 Department of Clinical Psychology and Psychotherapy, Trier University, Trier, Germany

This manuscript proposes a set of meta-analyses of individual patient data examining prognostic factors for treatment outcome in patients with depression. Prognostic factors include depressive symptoms, several indicators of disorder severity and a number of non-severity related factors. Databases were already searched for RCTs complying with the inclusion and exclusion criteria. Thirteen eligible RCTs were found, the protocol at hand describes the data analysis of the resulting 6271 patients.

 The proposed project is concerned with an important topic by aggregating the results of RCTs examining prognostic factors for treatment outcome. It addresses the problem of inconsistent findings due to different measures of disorder severity and their dependence on baseline symptom severity. This meta-analysis is a promising approach to elucidate the association between different prognostic factors and treatment outcome in depression.

 The hypotheses are derived comprehensibly from an extensive theoretical background. All supposed prognostic factors are based on recent empirical evidence or are otherwise well-founded. Instead of relying on an aggregated symptom severity score only, the authors capture the construct of disorder severity in a comprehensive manner based on several indicators.

 The reported methods are well described. The proposal deals with variable transformations and data preprocessing as well as sophisticated statistical analyses. To rule out confounding effects, potentially confounding variables are controlled individually.

We confirm that we have read this submission and believe that we have an appropriate level of expertise to confirm that it is of an acceptable scientific standard.

# Peer review: Reviewer response for version 1

**DOI:** 10.21956/wellcomeopenres.16615.r35663

**Published:** 2019-06-18

**Authors:** Julieta Galante

**Affiliations:** 1 Department of Psychiatry, University of Cambridge, Cambridge, UK

This article proposes a meta-analysis of individual participant trial data to explore prognostic factors of adult depression, and assess their influence independently of each other. The authors have already secured individual participant data for almost all of the trials to be included.

 I consider this manuscript to be well-written, with clear objectives and an appropriate study design. However, regarding reproducibility, I am not clear on the implication of the review search date, which is March 2019. I have to assume that data sharing agreements predate the search, which was only conducted to make sure no eligible trials were missing – was the 13 ^th^ trial a result of the search? It would be much clearer if the authors could spell out this process.

 Another confusing point is that the PROSPERO registration dated April 2019 shows Preliminary searches, Piloting of the study selection process, Formal screening of search results against eligibility criteria and Data extraction as ongoing or not started; this is inconsistent with what is stated in the manuscript. Similarly, in the Data handling and data management section the authors wrote “data from 13 studies were received…”. This is inconsistent with previous statements that data from one trial is pending.

 Figure 1 shows that pilot/feasibility trials were excluded (see records excluded). If these were RCTs and used a sample that was different from the larger trials that followed, it is unclear why they were excluded. If exclusion was pre-specified and performed in a systematic fashion, it should be explained and justified in detail as part of the exclusion criteria. If exclusion was not pre-specified systematically, then these trials should be included in the meta-analysis, or at least this mentioned as a limitation.

 Regarding generalisability, the discussion section reads: “findings from the Dep-GP database should be widely generalizable to adults with depression seeking treatment from their GP/family physician”. I find this too strong an assertion of generalisability - authors are only including trials from the UK, and there is cultural variability in many of the factors measured, so I would be more cautious regarding the external validity of the findings.

I confirm that I have read this submission and believe that I have an appropriate level of expertise to confirm that it is of an acceptable scientific standard, however I have significant reservations, as outlined above.

# Response

**Published:** 2019-10-24

**Authors:** Joshua Buckman

**Affiliations:** University College London, UK

*This article proposes a meta-analysis of individual participant trial data to explore prognostic factors of adult depression, and assess their influence independently of each other. The authors have already secured individual participant data for almost all of the trials to be included.*

*I consider this manuscript to be well-written, with clear objectives and an appropriate study design.*

**Response: **We thank Dr Galante for her thorough and informative review. In light of her comments we have made a number of amendments to the manuscript as detailed below.

*1. However, regarding reproducibility, I am not clear on the implication of the review search date, which is March 2019. I have to assume that data sharing agreements predate the search, which was only conducted to make sure no eligible trials were missing – was the 13th trial a result of the search? It would be much clearer if the authors could spell out this process.*

**Response: **Thank you for raising this important point, we have clarified our review process by adding the following to the manuscript:

Under the heading: **“Identification and selection of studies”:**

Searches were run in several stages, first an initial scoping search of the literature in November 2015, next a full search in April 2016 to identify studies and begin the process of data collection, then finally another search in March 2019 to ensure no studies published more recently were missed.

Under the heading **“Inclusion & exclusion criteria”:**

Inclusion and exclusion criteria were refined in the initial stage of the scoping searches for this work. After this refining process, studies were included if they were randomised clinical trials (RCTs) that had at least one active treatment arm and used the CIS-R at baseline to assess symptoms of anxiety and depression and to determine diagnoses. The studies were included if their samples were adults (aged 16 or over), with a diagnosis unipolar depression, depressive symptoms significant enough to lead them to seek treatment from their GP, or a CIS-R ( Lewis *et al*., 1992) score of ≥12, and if they were recruited from primary care centres.

Under the heading “ **Characteristics of the included studies”:**

In total, 15 RCTs were identified as meeting inclusion criteria for the IPD, of which 12 have provided individual patient-data and one is in the process of providing these data, see Figure 1. Nine studies were identified at the initial scoping stage, with a further three found in the second stage of searching and one final further study identified as meeting inclusion criteria after the final searches. After a consensus meeting once the final searches had been run, two studies previously considered to not meet inclusion criteria were re-classified as meeting criteria, and the study authors were contacted for IPD accordingly.

*2. Another confusing point is that the PROSPERO registration dated April 2019 shows Preliminary searches, Piloting of the study selection process, Formal screening of search results against eligibility criteria and Data extraction as ongoing or not started; this is inconsistent with what is stated in the manuscript.*

**Response: **At the time of writing we had just run our final searches, we were in the process of data collection and the final formal consideration of studies for inclusion and exclusion. As a part of that process we found one newly published study which met inclusion criteria and during a consensus meeting between the three reviewers involved in this process, we determined that two previously excluded studies would also have met inclusion criteria. As a result we contacted the authors of those latter two studies after submitting the initial version of the present manuscript, and we were informed that data from those studies were no longer available. So, it was accurate to state that the process of searching, the study selection process and data extraction were ongoing at the point of writing this manuscript and at the point of registration on PROSPERO. We will update our PROSPERO record as soon these processes are completed, once data from the final study are received and cleaned.

*3. Similarly, in the Data handling and data management section the authors wrote “data from 13 studies were received…”. This is inconsistent with previous statements that data from one trial is pending.*

**Response: **Thank you for pointing out this error, we have amended it to read:

“Data from the 12 trials were received and cleaned on an individual study basis before combining all studies into a single aggregated dataset, the final Dep-GP dataset will be formed once data from the 13 ^th^ study are received and cleaned”

*4. Figure 1 shows that pilot/feasibility trials were excluded (see records excluded). If these were RCTs and used a sample that was different from the larger trials that followed, it is unclear why they were excluded. If exclusion was pre-specified and performed in a systematic fashion, it should be explained and justified in detail as part of the exclusion criteria. If exclusion was not pre-specified systematically, then these trials should be included in the meta-analysis, or at least this mentioned as a limitation.*

**Response: **We have amended Figure 1 to show that 15 studies met our final inclusion criteria and the study authors from each of them were contacted for IPD. In terms of excluding the feasibility trials, this was part of our original pre-specified inclusion/exclusion criteria and was accordingly applied systematically across all searches.

*5. Regarding generalisability, the discussion section reads: “findings from the Dep-GP database should be widely generalizable to adults with depression seeking treatment from their GP/family physician”. I find this too strong an assertion of generalisability - authors are only including trials from the UK, and there is cultural variability in many of the factors measured, so I would be more cautious regarding the external validity of the findings.*

**Response: **We thank the reviewer for highlighting this, we have amended the manuscript accordingly, under the heading “ **Discussion and Conclusions**”:

The consistency in setting and the variability in both the populations drawn upon in the 13 studies and the treatments received in those studies means that findings from the Dep-GP database may be generalizable to adults with depression seeking treatment from their GP/family physician.
